# Radiation Therapy for Chordomas and Chondrosarcomas of the Skull Base: Evaluation of the Effectiveness of Treatment Methods (Review)

**DOI:** 10.17691/stm2023.15.5.05

**Published:** 2023-10-30

**Authors:** A.A. Lemaeva, I.A. Gulidov

**Affiliations:** Resident; A. Tsyb Medical Radiological Research Centre — Branch of the National Medical Research Radiological Centre of the Ministry of Health of the Russian Federation, 4 Koroleva St., Obninsk, 249036, Russia; MD, DSc, Professor, Head of the Radiotherapy Department; A. Tsyb Medical Radiological Research Centre — Branch of the National Medical Research Radiological Centre of the Ministry of Health of the Russian Federation, 4 Koroleva St., Obninsk, 249036, Russia

**Keywords:** chordoma, chondrosarcoma, skull base, radiation therapy, proton therapy

## Abstract

Chordomas and chondrosarcomas of the skull base are rare tumors. They are located in close proximity to critical structures, which poses a serious problem in the treatment of these tumors. Despite advances in surgery, radical resection is often not possible. Radiation therapy for chordomas and chondrosarcomas of the skull base is able to improve overall survival and local control.

**The aim of this review** is to analyze the literature data and evaluate the efficacy of radiation therapy techniques for chordomas and chondrosarcomas of the skull base. The most promising methods of radiation therapy for chordomas and chondrosarcomas of the skull base have been shown to be pencil-beam scanning proton therapy with intensity modulation and carbon ion therapy. These techniques have demonstrated high local control and overall survival with a low incidence of severe radiation-induced toxicity, which confirms their clinical benefits. It has also been found that stereotactic radiosurgery can be effectively used for small tumors (less than 7 cm^3^).

## Introduction

Chordomas and chondrosarcomas of the skull base are rare neoplasms characterized by slow, destructive, and locally invasive growth. They are often grouped together due to similarity of their anatomical localization, clinical manifestations, histopathological and radiographic signs, the character of growth, and prognosis.

Chordomas of the skull base make up less than 0.2% [1, 2] and chondrosarcomas — 0.15% of all intracranial tumors [3]. In the USA, about 350 cases of chordoma are registered annually [4], and 0.08 cases per 100,000 people worldwide [5]. These neoplasms occur equally frequently in men and women, usually between the ages of 50 and 60 years. Chordomas arise from the notochordal remnants. They are mainly localized in the regions of sacrum (50%), skull base (35%), and vertebral body (15%) [6]. The clivus of the occipital bone, the apex of the petrous temporal bone, and Meckel’s cave are the typical intracranial localizations. Chondrosarcomas originate from mesenchymal stromal cells or embryonic part of the cartilaginous matrix of the skull. Chondrosarcomas affects most commonly the bones of the axial skeleton (pelvis, scapula, sternum, and ribs) followed by the proximal femur and proximal humerus. Men have the disease 3 times more often than women do. These neoplasms develop commonly at the age of late forties. Chondrosarcomas of the skull base make up 7% of the total number of chondrosarcomas [7]. Their typical localization is the petroclival region and along the petro-occipital fissure.

Chordomas and chondrosarcomas are highly prone to local recurrences characterized by infiltrative growth with the destruction of the surrounding bone and soft tissues [8, 9]. These tumors usually demonstrate a slow character of growth and cause gradual displacement of neurovascular structures [10, 11]. Clinical manifestations are not typical and may vary significantly depending on the location, extension, and proximity of the damage to the critical structures [12, 13].

Computed tomography and magnetic resonance tomography are required to visualize the tumors. The majority of skull base chondrosarcomas are located laterally in contrast to chordomas which are usually positioned medially [14].

A modern concept of treatment represents a combination of surgical tumor resection and adjuvant radiation therapy. Chordomas and chondrosarcomas are not sensitive to chemotherapy, there are no approved chemotherapeutic agents to cure them [15, 16].

A complex structure of the skull base together with close proximity to the cranial nerves and vessels pose a serious problem for the treatment of these tumors. Radical resection with a negative surgical margin is usually difficult due to the aggressive and infiltrative character of tumor growth involving surrounding neurological structures [17, 18]. According to the literature data, the frequency of total resection varies from 0 to 60% [19, 20]. If total resection is not possible, partial resection is performed, which contributes to the improvement of general somatic status of a patient and to the reduction of the target volume for future radiotherapy.

**The aim of the present review** is to analyze the literature data and evaluate the effectiveness of radiation methods of therapy for chordomas and chondrosarcomas of the skull base.

The searches of literature were performed using databases Scopus, Web of Science, PubMed, Science Direct, Google Scholar by the following key words: chordoma, chondrosarcoma, skull base, radiation therapy, proton therapy, stereotactic radiation therapy, radiosurgery, ion therapy, surgery.

## Radiation therapy. Treatment techniques

Chordomas and chondrosarcomas are radioresistant tumors and require high doses of radiation therapy. In addition, it is necessary to optimize the dose distribution in complex target volumes, following the constraints of the nearest critical structures. For chordomas, a clear dose-effect dependence, in which the best local tumor control may be achieved in patients receiving more than 75 Gy, has been demonstrated, whereas at the total focal dose from 40 to 60 Gy, the control is achieved only in 20% of cases [21].

Radiation therapy may be used as an independent option of treating unresectable tumors, as well as in the adjuvant mode after resection. Adjuvant radiation therapy is able to improve the local control and overall survival. In the systematic literature review [22], it has been shown that surgical treatment supplemented by any form of radiation therapy in patients with intracranial chondrosarcoma reduced five-year lethality from 25 to 9%.

If photon beam radiation therapy is used, a high-dose irradiation is, as a rule, impossible due to the existing limitations of the dose burden on the critical structures (visual nerves, chiasma, brainstem, spine, cerebrum, and other structures). There are reports in the literature on five-year local control and progression-free survival rate within the range of 15-66%. These results speak of the fact that even modern improved methods of photon irradiation fail in many cases to achieve adequate delivery of the radical doses to the target not exceeding the level of critical structure tolerance for the tumors localized in the skull base [23].

Generally, the application of large fractions cannot solve this problem (Table 1). For example, Martin et al. [24] reported a low five-year level of local control, which amounted to 53% after a single procedure of stereotactic radiosurgery for treating skull base chordomas. Other authors have published similar results [25, 26].

**T a b l e 1.** Stereotactic radiosurgery for treating chordomas (CA) and chondrosarcomas (CSA) of the skull base

References	Number of patients	Average tumor volume, min-max (cm^3^)	Average administered TFD, min-max (Gy)	5-year local control (%)	5-year overall survival (%)	Toxicity
Martin et al. [24]	28	9.8 (0.078-22.0)	16.0 (10.5-25.0)	CSA — 80 CA — 62.9	CSA — 88 (over 6 years) CA — 53.4	Radionecrosis in 3 patients (10.7%)
Krishnan et al. [25]	29	14.4 (0.6-65.1)	15 (10-20)	CSA — 100 CA — 32	CSA — 100 CA — 32	Complications (34%) included deficit of cranial nerves (n=6), radiation necrosis (n=5), and hypophysis dysfunction (n=3)
Hasegawa et al. [26]	37	22.0 (0.4-94.3)	14 (9-20)	CSA and CA — 76	CSA and CA — 80	Neurological deficit in one patient (0.3%)
Cahill et al. [27]	24	10 (1-36)	20 (13-25)	CSA — 78 CA — 67	CSA — 78 CA — 67	There was no toxicity grade III or higher

N o t e: TFD — total focal dose.

At the same time, stereotactic radiosurgery represents a sufficiently effective variant of treating small-sized tumors. Thus, in the study conducted in Great Britain [27], 15 patients in the chordoma group and 9 patients in the chondrosarcoma group with an average tumor volume of 13 and 12 cm^3^, respectively, underwent stereotactic radiosurgery with a gamma knife. Five- and ten-year overall survival in the chordoma group was 67 and 53%, respectively, whereas in the chondrosarcoma group it was 78% for both indicators. The indicators of local tumor control after 5 and 10 years in the chordoma group were 67 and 49%, respectively, and in the group with chondrosarcoma — 78% for both periods. The tumor size under 7 cm^3^ was associated with a higher overall survival. The indicators of the overall survival rate and local control in this study are comparable with the results of treatment with a proton beam.

A large retrospective analysis [28] has demonstrated that an application of proton therapy (relative to photon therapy) at the dose exceeding 70 Gy is a predictor of improvement of 5-year overall survival. It has also been revealed in the study [29] that the usage of proton therapy may reduce the radiation dose to the adjacent healthy tissues by about 50% compared to photon beams.

Protons and carbon ions have a physical advantage over photons. The dose distribution in the tissue begins with low values at the beam entrance. Later, the dose grows with the increase of the penetration depth. At the end of the particle’s path, a sharp maximum is observed and the Bragg peak is formed. Then, the dose drops to zero within a few millimeters. Owing to the presence of the Bragg peak and an insignificant scattering on the path to the exposed target, the following advantages may arise: the possibility of dose concentration within the target volume, i.e. at the end of the particle path; minimization of the dose in the surrounding tissues; the possibility to adjust the position of the length of the dose maximum; almost complete absence of radiation scattering; high marginal gradient of the dose; insignificant radiation burden to the tissues situated beyond the pathological focus along the path of the proton or ion beam [30].

Proton therapy in chordomas and chondrosarcomas of the skull base is applied in the mode of traditional functioning, which is connected with close position of the tumor to the critical structures. Two main methods of radiation are used: passive scattering and a more advanced method — active scanning proton therapy.

Until recently, passive scattering has been the most widely used method of proton radiation therapy, in which a beam of protons is distributed in the space by a scattering foil, is shaped by an aperture just as it is done in 3D conformal photon therapy. Depth distribution in this case is modulated with compensators. In comparison with the active scanning beam, passive scattering technique is presently technically obsolete as it shows the worst characteristics of dose distribution, requires the preparation of field forming devices, and generates secondary neutrons [31]. When protons collide with the scattering material and beam forming devices, they lose energy or reduce the radiation range necessary for treating the patient. It is more difficult to deliver an optimal dose to the focus [32].

In 1980, Kanai et al. [33] proposed a system of spot scanning for proton radiation therapy. This technique was further developed in the Paul Scherrer Institute [34], where in 1995 spot scanning proton beam therapy was used for the first time. The possibilities of the spot scanning technique were further expanded using intensity-modulated proton therapy (IMPT) [35-37]. At present, IMPT is the most precise and advanced type of proton therapy.

Now, proton therapy with a thin pencil-beam scanning (PBS) is a cutting-edge method of cancer treatment employed in a few proton centers in the world (mainly in the USA, West Europe, and Japan [38]). The technology of active intensity-modulated scanning is based on magnetic properties of the particles. A thin beam of protons with individual energy necessary for reaching the depth of tumor location is generated in a cyclotron or synchrotron. The beam path is deflected by the magnets, and protons gradually fulfil the entire volume of target irradiation [32].

Dosimetric studies have confirmed that application of intensity modulated proton therapy (IMPT) with the technology of pencil-beam scanning (PBS) and intensity modulated carbon-ion technology (IMCT) may improve the coverage of the target volume with minimization of the dose for the surrounding structures at risk, enhancing thereby a therapeutic effect for the skull base tumors [39].

Historically, chordomas and chondrosarcomas of the skull base became the first targets for proton therapy. In 1999, the first experience of two clinical settings, which implemented proton radiotherapy in the USA, was was presented [40, 41] (Table 2). The first large investigation was conducted in the Massachusetts General Hospital (Boston, USA) [40] and included 519 cases of chordomas and chondrosarcomas of the skull base. Patients received courses of proton and photon therapy at a total focal dose (TFD) from 66 to 83 GyRBE. A follow-up period was from 1 to 254 months (median 41 months). Recurrence-free 5-year survival rate appeared to be higher for chondrosarcomas than for chordomas: 98 vs 73% and 94 vs 54% after 10 years, respectively. The overall survival rate was also higher in patients with chondrosarcomas than in those with chordomas: 91 vs 80% after 5 years and 88 vs 54% after 10 years, respectively. The frequency of severe complications was noted to be low. There were registered 3 cases (0.6%) of death due to brainstem damage, 8 cases (1.5%) of temporal lobe injury, 12 cases (2.3%) of optical neuropathy, 15 cases (2.9%) of hearing loss (in 2/3 of patients, who received 62.7 GyRBE and more to the cochlear or auditory nerve, severe hearing loss was revealed), 32 cases (6.2%) of endocrinopathies. The high local control rates of proton therapy have been confirmed in other studies as well.

**T a b l e 2.** Proton therapy for treating chordomas (CA) and chondrosarcomas (CSA) of the skull base

References	Number of patients	Radiation therapy	Administered TFD, min-max (GyRBE)	5-year local control (%)	5-year overall survival (%)	Toxicity
Feuvret et al. [19]	159	Protons + photons	61-71	CSA — 96.4	CSA — 94.9	Indicators of toxicity grade III—V after 5 and 10 years were 10%
Munzenrider and Liebsch [40]	519	Protons + photons	66-83	CA — 73 CSA — 80	CA — 80 CSA — 91	3 cases (0.6%) of death due to brainstem damage, 8 cases (1.5%) of temporal lobe injury, 12 cases (2.3%) of optical neuropathy, 15 cases (2.9%) of hearing loss, 32 cases (6.2%) of endocrinopathy
Hug et al. [41]	58	PBS, PSPT	65-79	CA — 76 CSA — 92	CA — 79 CSA — 100	Late toxicity grade III and IV in 4 patients (7%)
Ares et al. [42]	64	PBS	67-74	CA — 81 CSA — 94	CA — 62 CSA — 91	Late toxicity of high severity grade in 4 patients (6.25%)
Parzen et al. [43]	13	PBS	70.0-75.8	CSA and CA — 100	CSA and CA — 100	There was no toxicity grade III or higher
Holtzman et al. [44]	112	Protons + photons	69.6-74.4	CA — 74	CA — 78	There was no toxicity grade III or higher. Necrosis of temporal lobe grade II developed in 1 patient (0.9%)
Gordon et al. [45]	31	PBS	70	CSA and CA — 85.3 (3 years)	CSA and CA — 66.3 (3 years)	2 cases (6.5%) of toxicity grade >III including 1 case (3.2%) of grade III myelitis and 1 case (3.2%) of brainstem injury grade V

N o t e: TFD — total focal dose, PBS — pencil-beam scanning, PSPT — passive scattered proton therapy.

The study performed in the Loma Linda University Medical Center [41] presented a successful experience of proton therapy after surgical resection in 58 patients. After various surgical interventions, a residual tumor was identified in 91% of patients. A total focal dose to the target area was 65-79 GyRBE. Proton therapy was used with a fixed spot beam and movable gantry systems. The follow-up period lasted from 7 to 75 months (median 33 months). Five-year local control was 92% for chondrosarcomas and 76% for chordomas. The tumor volume and involvement of the brainstem influence the indicators of local control. All tumor volumes of 25 ml or less remained locally controllable; local recurrences occurred in 56% of cases with a tumor size over 25 ml. No recurrences were found in 94% of patients without brainstem affection; in patients with the involved brainstem (and reduction of the dose due to the limited tolerance of the brainstem), tumor control was achieved only in 53% of cases. The overall five-year survival rate was 100% for patients with chondrosarcoma and 79% with chordoma. Late toxicity grade III and IV was observed in 4 patients (7%) and was accompanied by symptoms in 3 individuals (5%).

In 2009, Ares et al. [42] reported the results of using proton therapy by pencil-beam scanning in 64 patients. Patients with skull base chordoma received an average total dose of 73.5 GyRBE (range: 67-74 GyRBE) to the target region, whereas the dose for patients with chondrosarcoma was 68.4 GyRBE (range: 63-74 GyRBE). At a median follow-up of 38 months, 5-year indicators of local control amounted to 81% for chordomas and 94% for chondrosarcomas. The overall five-year survival rate was 62% for patients with chordoma and 91% with chondrosarcoma. Brainstem compression and gross tumor volume (GTV) more than 25 cm^2^ were noted to be predictors of the lower local control indicators. Late toxicity of a high severity grade was observed only in four patients.

Later in 2014, Grosshans et al. [46] published the results of using proton therapy for treatment of 15 patients with chordomas and chondrosarcomas of the skull base. Average administered radiation doses were 69.8 GyRBE (range: 68-70 GyRBE) for chordomas and 68.4 GyRBE (range: 66-70 GyRBE) for chondrosarcomas. In comparison with the plans of passive scattering, those of the spot scanning have demonstrated better conformity at high TFD and provided less dosage to the region of the temporal lobes and brainstem. Only one case of local recurrence and one case of distant metastasis were reported; no cases of severe toxicity at a median follow-up period of 27 months were registered.

In 2016, Feuvret et al. [19] reported the results of treating 159 patients with skull base chondrosarcoma receiving the combination of photon and proton therapy with a fixed horizontal beam (n=126) or using mobile gantry system (n=23). When delivering radical doses to the target region (average TFD 70.2 GyRBE), the authors achieved good results: five- and ten-year survival rates were 94.9 and 87%, respectively, whereas the level of toxicity was low at a median follow-up period of 77 months. Late toxicity grade III-V over 5 and 10 years amounted to 10% for both periods of follow-up. The age below 40 years, primary form of the disease, and tumor volume less than 18 cm^3^ have been also found to be the prognostic factors increasing the indicators of overall survival and progression-free survival. In contrast, the operation volume, dosimetric parameters, and proximity of critical structures did not influence the values of local control and overall survival.

In 2021, the researchers from the USA [43] reported a successful experience of treating chordomas and chondrosarcomas of the skull base in 13 patients using PBS technology concurrently with the method of simultaneous integrated boost to increase conformity and reduce the dose to the critical structures in comparison with the sequential increase of the radiation volume. An average dose to GTV was equal to 72.4 GyRBE (range: 79.0-75.8 GyRBE). An average GTV was 3.4 cm^3^ (range: 0.2-38.7 cm^3^). Toxicity grade III or higher was not observed. Necrosis of the temporal lobe grade II developed in one patient. The indicators of the local control and overall survival rate were 100% at a median follow-up period of 10.7 months.

The study performed by Holtzman et al. [44] also proved the efficacy of the postoperative proton therapy with high doses for chordomas. The study included 112 patients, of which 105 (94%) received proton and 7 (6%) — proton-photon therapy in the period from 2007 to 2019. On average, 73.8 GyRBE was delivered to the target area (range: 69.6-74.4 GyRBE). Five years after radiation therapy, the overall survival was 78%, 5-year local control was 74%. Average time to the local recurrence was 2.4 years (range: 0.8-7.0 years). Severe radiation damages were not noted at a median follow-up period of 4.4 years (range: 0.4-12.6 years).

The study conducted in the A. Tsyb Medical Radiological Research Centre (Obninsk, Russia) [45] has analyzed 31 cases of treating chordomas and chondrosarcomas of the skull base. Modulated intensity proton therapy was performed using a fixed horizontal pencil-beam scanning with a patient in a sitting position. An average total dose was 70 GyRBE. An average tumor volume amounted to 25.6 cm^3^. A median follow-up period was 21 months, besides the authors reported on good indicators of two- and three-year local control (93 and 85.3%, respectively) and also on a low level of toxicity. Progression in the cervical lymph nodes and lungs was observed in two patients with chondrosarcoma. Two cases of severe toxicity were revealed including one case of myelitis grade III and one of brainstem injury grade V.

Nie et al. [47] have published a systematic review of the clinical experience of using proton therapy for treating chordomas and chondrosarcomas. The analysis includes seven investigations, which involved 478 patients. The follow-up period lasted within the range of 21.0-61.7 months. When planning proton therapy, an average target volume was from 15 to 40 cm^3^, while TFD varied from 63.0 to 78.4 GyRBE. High indicators of 5-year local control (78%) and overall survival (85%) at a low frequency of severe radiation-induced toxicity have been noted. This paper confirms again a relatively high effectiveness and low toxicity of proton therapy.

The experience of using carbon ion therapy for chordomas and chondrosarcomas of the skull base is also presented in the literature; the results of both technologies are quite comparable (Table 3).

**T a b l e 3.** Ion therapy for treatment of chordomas (CA) and chondrosarcomas (CSA) of the skull base

References	Number of patients	Radiation therapy	Administered TFD (GyRBE)	5-year local control (%)	5-year overall survival (%)	Toxicity
Schulz-Ertner	96	Carbon ions	60	CA — 70	CA — 88.5	Visual nerve neuropathy
et al. [21]						RTOG/EORTC grade III in 4.1% of cases and adipose fold necrosis in 1 patient
Mizoe et al. [48]	19	Carbon ions	60.8	CA — 100	CA — 100	There was no toxicity grade III or higher
Uhl et al. [49]	155	Carbon ions	60	CA — 72	CA — 85	There was no toxicity grade III or higher
Guan et al. [50]	91	Protons (P) and/or carbon ions (I)		CA and CSA (2 years) P+I: 86.2 P/I: 86.7	CA and CSA (2 years) P+I: 87.2 P/I: 93.8	Acute mucositis grade III in 1 patient (1%)
Mattke et al. [51]	111	Carbon ions	66	CA — 65	CA — 83	Temporal lobe necrosis in 20 patients (13.6%) with left majority of cases being symptom-free (toxicity grade I) or responding well to therapy with steroids or bevacizumab (toxicity grade II-III)
	36	Protons	74	CA — 61	CA — 92	
Iannalfi et al. [52]	112	Carbon ions	70.4 (SFD — 4.4)	CA — 71	CA — 82	There was no toxicity grade III or higher
		Protons	74	CA — 84	CA — 83	

N o t e: TFD — total focal dose, SFD — single focal dose.

In the study conducted in Japan [48], a median follow-up amounted to 53 months. No late toxicity of the severe degree has been noted over this time. Nineteen patients with skull base chordoma, who received 60.8 GyRBE, were noted to show excellent results; 5-year local control and overall survival rates were 100%.

Schulz-Ertner et al. [21] have analyzed the results of carbon ion therapy for chordomas. All patients (n=96) had large-volume residual tumors: an average volume was 80.3 (13.9-594.2) ml. An average TFD was equal to 60 GyRBE. The administered total doses to the tumor exceeding 60 GyRBE and the status of the primary tumor were associated with higher values of local control. Five-year overall survival was 88.5% and local control was 70% at a median follow-up period of 31 months. Late toxicity was noted in 4.1% of cases in the form of visual nerve neuropathy (RTOG/EORTC grade III) and adipose fold necrosis in one patient. Insignificant damage to the temporal lobe (RTOG/EORTC I-II grade) was observed in seven patients (7.2%).

In Germany, a total number of 155 patients with chordoma of the skull base received radiation therapy with carbon ions using a raster scanning technique in the period from 1998 to 2008 [49]. An average TFD was 60 GyRBE at a single dose of 3 GyRBE. The target volume was in the range from 2 to 294 ml (average 70 ml). Five- and ten-year indicators of local control were 72 and 54%, respectively, while the overall survival rate was 85 and 75%, respectively. No late toxicity of severe grades has been noted.

The comparison of proton and ion therapy for chordomas and chondrosarcomas of the skull base was presented in the study conducted in China in 2019 [50]. Of the 91 patients, 8 received only proton therapy, 28 — combined proton and carbon-ion therapy, 55 were treated only with carbon ions. An average total tumor volume was 37 cm^3^. At a median follow-up of 28 months, two-year local control, recurrence-free, and overall survival were 86.2, 76.8, and 87.2%, respectively. Proton and ion therapy showed comparable results for toxicity, local control, and overall survival, although the follow-up period was not long. The multifactorial analysis has demonstrated that the tumor volume exceeding 60 cm^3^ served as a single significant factor for predicting the event-free survival, whereas re-irradiation and a tumor volume over 60 cm^3^ were significant prognostic factors for overall survival. Late toxicity grade I-II was observed in eleven patients, one developed acute mucositis grade III.

In 2022, the study carried out in the Heidelberg Ion Beam Therapy Center (Germany) also showed comparable results of treating skull base chordomas with protons and carbon ions [51]. Of 147 patients receiving the therapy, 111 were treated with carbon ions, 36 — with protons. An average dose to the target was 66 GyRBE for ion therapy and 74 GyRBE for proton one. At the beginning of the radiation therapy, brainstem compression was observed in 38% of patients, contact position without compression of the brainstem — in 18% of individuals, location at a distance of less than 3 mm from the brainstem — in 16% of patients. At a median follow-up period of 49.3 months, a local recurrence was found in 41 patients (27.9%). Significant differences between proton and carbon ion therapy in relation to overall survival, local control, or general toxicity have not been observed. The indicators of local control over 1 year, 3 years, and 5 years were 97, 80, 61% (protons) and 96, 80, 65% (carbon ions), respectively. The overall survival rates were 100, 92, 92% (protons) and 99, 91, 83% (carbon ions). Acute radiation reactions such as mucositis or skin toxicity were, as a rule, moderate and similar in the groups treated with carbon ions and protons. A total of 44 patients were revealed to have radiation reactions in the temporal lobe. Necrosis of the temporal lobe, identified in 20 of these patients, became the most severe late toxic effect. The majority of cases were symptom-free (toxicity grade I) or responded well to steroid/bevacizumab therapy (toxicity grade II-III). On the whole, acute or late toxicity grade IV or lethal outcomes related to the treatment were not observed. The results of treatment of skull base chordomas with protons and carbon ions seems to be similar in regard to tumor control, survival rate, and toxicity.

In the prospective study of the National Center for Oncological Hadrontherapy (Italy) [52], ion therapy was used at a larger single dose (4.4 GyRBE per fraction) and was administered to the patients with a higher risk, whereas proton therapy for skull base chordomas was used at a dose of 2 GyRBE per fraction — to treat patients with a lower risk (a small residual tumor volume). A median follow-up period was 49 months. The study has demonstrated that the choice of the radiation type (protons or carbon ions) did not influence the indicators of local control (p=0.15) and overall survival rate (p=0.82). In the group of ion therapy, indicators of 3- and 5-year local control were equal to 77 and 71%; the indicators of overall survival over the same periods were 90 and 82%, respectively. In the group of proton therapy, the indicators of 3- and 5-year local control were equal to 89 and 84%, the overall survival rates over the same periods were 93 and 83%, respectively. Acute toxicity grade III or higher was not observed. Compression of the brainstem, residual tumor volume, and coverage of the target volume were the main prognostic factors of local control.

At present, chordomas and chondrosarcomas of the skull base are presented in the recommendations of NCCN and ESMO as preferable targets for proton and carbon ion therapy, however, these methods are noted to require further exploration and active implementation in clinical practice [53, 54]. Unfortunately, the availability of these technologies is yet insufficient, especially in the developing countries.

## Conclusion

Radiation therapy for chordomas and chondrosarcomas of the skull base is a challenging clinical task. The most promising techniques of radiation therapy for these neoplasms are pencil-beam scanning intensity-modulated proton therapy and carbon ion therapy. These technologies have shown high local control and survival rate at a low frequency of severe radiation-induced toxicity, which confirms their clinical benefits. It has been also established that stereotactic radiosurgery may be effectively used for small tumors. However, these technologies need further exploration: a deeper analysis focused on prognostic factors, interdisciplinary planning, and optimization of the treatment methods are required.
